# Lagrange Programming Neural Network for TOA-Based Localization with Clock Asynchronization and Sensor Location Uncertainties

**DOI:** 10.3390/s18072293

**Published:** 2018-07-15

**Authors:** Changgui Jia, Jiexin Yin, Ding Wang, Li Zhang

**Affiliations:** 1 National Digital Switching System Engineering and Technology Research Center, Zhengzhou 450002, China; Kingsway0221@163.com (C.J.); wang_ding814@aliyun.com (D.W.); neyou1@163.com (L.Z.); 2 Zhengzhou Information Science and Technology Institute, Zhengzhou 450002, China

**Keywords:** source localization, time-of-arrival (TOA), clock asynchronization, sensor position uncertainties, Lagrange programming neural network (LPNN), analog neural network

## Abstract

Source localization based on time of arrival (TOA) measurements in the presence of clock asynchronization and sensor position uncertainties is investigated in this paper. Different from the traditional numerical algorithms, a neural circuit named Lagrange programming neural network (LPNN) is employed to tackle the nonlinear and nonconvex constrained optimization problem of source localization. With the augmented term, two types of neural networks are developed from the original maximum likelihood functions based on the general framework provided by LPNN. The convergence and local stability of the proposed neural networks are analyzed in this paper. In addition, the Cramér-Rao lower bound is also derived as a benchmark in the presence of clock asynchronization and sensor position uncertainties. Simulation results verify the superior performance of the proposed LPNN over the traditional numerical algorithms and its robustness to resist the impact of a high level of measurement noise, clock asynchronization, as well as sensor position uncertainties.

## 1. Introduction

Source localization is an essential task in radar, sonar, navigation, and other applications [1,2,3,4]. Generally, the process of source localization involves two stages. At the first stage, a network of collaborative signal sensors is deployed in the space to obtain a collection of distinct signal measurements from the signal emitters. These measurements are then transmitted to the Data Fusion Center to generate an estimate of the source coordinates at the second stage. According to the authors of [1,4,5], among different data fusing methods, time of arrival (TOA) measurements can be utilized to locate a source with high accuracy. In addition, the single source positioning problem considered in this paper is based on the basic assumption that the received TOA measurements have been matched with the original emitting source, and the relevant association methods can be found in [6,7].

In the TOA model, the distance between the source and sensor is obtained by measuring the signal propagation time from source to sensor. Through the conventional modeling method with several sensors, the source position is determined by the intersection of a set of circles [8]. Because of the measurement noise, the TOA measurements are usually contaminated, and thus we can only obtain an intersection of area that a source could lie within.

Apart from the measurement noise, in many emitting source localization scenarios, clock asynchronization between source and sensors is a quite common problem [9], which will also make it difficult to obtain real TOA measurements. Under such non-cooperative situations, the start transmission time is not known at the receiving sensors, thus causing a common time offset among all the received TOA measurements due to uncertainty of the start transmission time instant $t_{o}$. Unfortunately, this uncertainty will definitely impose localization errors when simply assuming $t_{o}=0$ in modeling.

To tackle the asynchronization problem, several joint synchronization and localization numerical algorithms utilizing TOA measurements have been put forward. They are mainly divided into two approaches, namely iterative algorithms [10,11] and close-form algorithms [12,13]. In [10], the author develops an iterative maximum-likelihood (ML) algorithm based on Taylor-series expansion. However, this iterative ML algorithm requires fine initial estimates for the global solution. Otherwise, it may suffer from the problem of local convergence. Recently, the authors of [11] extended this methodology to a more-sophisticated scenario where source velocity and sensor clock offset are considered, and an efficient and robust ML algorithm is employed. The authors in [12,13] propose two types of weighted least square (WLS) based low-complexity algorithms, respectively, which can approach the LS solution in estimation performance at small noise conditions. Alternative approaches jointly estimate the source position and the start transmission time by utilizing semidefinite relaxation (SDR) [5,14] and second-order cone program (SOCP) [15] techniques based on convex optimization theory. However, they are not optimal to achieve the Cramér-Rao lower bound (CRLB) and it is also not a trivial task to reformulate the original non-convex optimization problem into a convex one.

In this article, a neural network framework called the Lagrange programming neural network (LPNN) [16] is employed to the localization problem. Since the first Hopfield network [17] was proposed to solve various optimization problems, it has received a lot of attention to construct and use neural circuits for optimization [18,19]. Different from conventional numerical algorithms running on computers, analog neural circuits make use of the operational advantage of hardware circuits to fulfill real-time calculations. Generally, neural circuits can be realized by large-scale integration (VLSI) or optical technologies. To sum up, the neural circuits provide new ideas to solve various optimization problems, especially when the computing resource is limited in some applications.

Among the existing neural circuits, the LPNN is capable of solving many types of optimization problems with constraints [16]. Recently, the LPNN method was applied in waveform design [20], MIMO radar [2] and source localization [21,22,23]. For example, the authors of [21,22] built two robust LPNN using TOA measurements in ideal localization scenarios. This methodology was then extended to the TDOA model [23]. These preliminary works exhibit the excellent performance of LPNN and reference herein [21,22,23].

This paper deals with the TOA-based localization problem by utilizing LPNN framework in the presence of clock asynchronization. It is then extended to a scenario where sensor positions are not exactly known. We note that the extension is necessary as sensor position uncertainties can dramatically deteriorate localization performance [14] in some scenarios. Unlike other LPNN-based articles, our paper considers a more complex TOA-based positioning scene where clock synchronization is not achieved and the sensor positions are not accurately known. This sets our work apart from other existing literature utilizing LPNN for localization problem. Our simulation results demonstrate that the proposed LPNN method can obtain CRLB in a wide measurement noise range and it is also robust enough to resist the impact of large clock offset and sensor position uncertainties. The contributions of this paper can be summarized as follows.

(1) The ML-based TOA localization problem in a non-ideal environment is formulated as a constrained optimization problem, which can be solved by the LPNN, and the inequality constraints are also transferred to equalities with the aid of additional variables and constant multipliers.
(2) We then develop two stable LPNN models by adding the augmented term to improve the convexity of objective function when synchronization is not achieved and the sensor positions are erroneous.
(3) The convergence and stability of the two LPNN models are analyzed in this paper. Besides, simulations are also performed to illustrate their convergence and stability.

The rest of the paper is organized as follows. A brief introduction of LPNN and the TOA measurement model with clock asynchronization are given in Section 2. Subsequently, the LPNN method for solving the localization problem is presented in Section 3. Then, the method is extended to the case with sensor position uncertainties in Section 4. Section 5 discusses the convergence and stability analysis of the proposed two neural networks. Section 6 derives the explicit CRLB in the presence of time asynchronization using TOA measurements, as well as the case with sensor position uncertainties. Numerical simulation results are illustrated in Section 7. Finally, we conclude our work in Section 8.

## 2. Problem Formulation

### 2.1. Review on LPNN

Generally, consider the nonlinear programming problem with equality constraints given by:

(1) $$\begin{array}{l} \underset{x}{\min}\;f(x)\\ s.t.\;h(x)=0_{m\times 1} \end{array},$$

where $x={\left(x_{1},x_{2},\ldots ,x_{n}\right)}^{T}\in {\mathbb{R}}^{n}$ denotes the vector of optimization variable. $f:{\mathbb{R}}^{n}\to \mathbb{R}$ represents the objective function which is nonlinear. $h:{\mathbb{R}}^{n}\to {\mathbb{R}}^{m}\;(m\leq n)$ is the constraint function that describes the m equality constraints and $0_{m\times 1}$ denotes m×1 zero vector. Moreover, f and h are further required to be twice continuous differentiable.

The Lagrangian function exploited in the LPNN approach is formulated as:

(2) $$L(x,\lambda )=f(x)+\lambda^{T}h(x),$$

where $\lambda \in {\mathbb{R}}^{m}$ represents the Lagrange multiplier vector. Generally, there are two types of neurons, namely variable neurons and Lagrangian neurons, in the LPNN model. The optimization variables x is held in the variable neurons and the Lagrange multipliers λ is held in the Lagrangian neurons. Implemented in an analog way, these two types of neurons in the LPNN work collaboratively to search an equilibrium point of (2) and the transient behavior of the neural network is governed by the following dynamics:

(3) $$\begin{array}{l} \frac{dx}{dt}=-\nabla_{x}L(x,\lambda ),\\ \frac{d\lambda }{dt}=\nabla_{\lambda }L(x,\lambda ), \end{array}\;$$

where t is the time variable. The differential equations in (3) further indicate that the dynamics of optimization variables x will decrease the Lagrangian function, while the optimization variables x are constrained by the dynamics of Lagrange multipliers to meet the constraints. When the network converges and eventually stabilizes to an equilibrium point in the LPNN model, as proved in [16], this equilibrium point satisfies the necessary KKT condition for the optimal solution of the nonlinear programming.

### 2.2. TOA-Based Source Localization with Time Asynchronization

In the TOA model, there are M (M≥3) sensors deployed in the space to collaboratively locate an emitting source with unknown position $u={\left[u_{1},u_{2}\right]}^{T}$. Let $s_{i}^{\circ }={\left[s_{i,1}^{\circ },s_{i,2}^{\circ }\right]}^{T}$ i=1,2,…,M denote the true coordinate vector of the i-th sensor. Without loss of generality, the line-of-sight propagation (LOS) is assumed and the local clocks of the collaborative sensors are synchronized. Therefore, under the condition of source-sensor asynchronization, the TOA measurement from the source to the i-th sensor is computed as:

(4) $$t_{i}=\frac{1}{c}\left\|u-s_{i}^{\circ }\right\|+t_{o}+e_{i},\;i=1,2,\ldots ,M$$

where ‖·‖ stands for the Euclidean norm and c is the signal propagation speed. $t_{o}$ is the unknown clock instant at which the source transmits the signal. $e_{i}$ is measurement noise, which is assumed to be zero-mean Gaussian distributed with variance $\sigma_{e}^{2}$. By multiplying c and the TOA measurements, the distance between i-th receiver and the source is denoted as:

(5) $$d_{i}=\left\|u-s_{i}^{\circ }\right\|+\delta_{o}+n_{i}\;i=1,2,\ldots ,M$$

where $\delta_{o}=c\cdot t_{o}$, $n_{i}=c\cdot e_{i}$ and $E(n_{i}\cdot n_{i})=\sigma_{i}^{2}=\sigma^{2}$. For simplicity, we assumed that the measurement noise powers are identical at all sensors as [15,21]. Based on (5), the joint probability density function (PDF) of the range measurements is:

(6) $$p\left(d_{1},d_{2},\ldots ,d_{M}|u,\delta_{o}\right)={\left(2\pi \sigma^{2}\right)}^{-\frac{M}{2}}\exp\left(-\frac{1}{2\sigma^{2}}\sum_{i=1}^{M}{\left(d_{i}-\left\|u-s_{i}^{\circ }\right\|-\delta_{o}\right)}^{2}\right).$$

In the TOA-based localization, given the measurements $d={\left[d_{1},d_{2},\ldots ,d_{M}\right]}^{T}$ and the coordinates of sensors, the source position can be estimated through minimizing the following log-likelihood function:

(7) $$\left\{\hat{u},{\hat{\delta }}_{o}\right\}=\arg\;\underset{u,\delta_{o}}{\min}\frac{1}{2\sigma^{2}}\sum_{i=1}^{M}{\left(d_{i}-\left\|u-s_{i}^{\circ }\right\|-\delta_{o}\right)}^{2}.$$

The problem of interest is to jointly estimate the source position and the source clock bias given the sensor positions and noisy TOA measurements. It is not a trivial task to solve $\hat{u}$ in (7) since the objective function is a nonlinear and nonconvex function of u. Thus, in Section 3, the LPNN is employed to tackle this problem. We point out that this article focuses on source localization and synchronization problems in a two-dimensional plane, and the result can be generalized straightforwardly to the three-dimensional scenario.

## 3. Proposed Method

For clarity, we recast the ML estimation problem in (7) as a constrained optimization problem:

(8) $$\begin{array}{l} \underset{u,g,\delta_{o}}{\min}\frac{1}{2\sigma^{2}}\sum_{i=1}^{M}{\left(d_{i}-g_{i}-\delta_{o}\right)}^{2}\\ s.t.\;g_{i}^{2}={\left\|u-s_{i}^{\circ }\right\|}^{2}\;,\\ \;g_{i}\geq 0,\;i=1,2,\ldots ,M \end{array}$$

Since (8) contains inequality constraints, we introduce the additional variables $y_{i},\;i=1,2,\ldots ,M$ to transform the inequality constraints into equalities and fit the equality constrained problem illustrated in (1). Our aim is to ensure that $g_{i}$ is non-negative, thus it is straightforward to let $g_{i}=\alpha_{i}\cdot y_{i}^{2}$. Here, $\alpha_{i},\;i=1,2,\ldots ,M$ are constants and $\alpha_{i}\geq 1$, which are utilized to accelerate the convergence [16]. Note that any differentiable positive function of $y_{i}$ confined within proper dynamic range, is also suitable to achieve this aim [16] and $y_{i}^{2}$ is employed for simplicity. The problem is then formulated as:

(9) $$\begin{array}{l} \underset{u,g,\delta_{o}}{\min}\frac{1}{2\sigma^{2}}\sum_{i=1}^{M}{\left(d_{i}-g_{i}-\delta_{o}\right)}^{2}\\ \;s.t.\;g_{i}^{2}={\left\|u-s_{i}^{\circ }\right\|}^{2}\;,\\ \;g_{i}=\alpha_{i}\cdot y_{i}^{2},\;i=1,2,\ldots ,M \end{array}$$

Notice that the objective function contained in (9) are twice differentiable, as well as the constraints. Thus, the LPNN framework is able to be applied to tackle the constrained optimization problem in (9). According to (2), the Lagrangian function is calculated as:

(10) $$L_{c}\left(u,g,\delta_{o},y,\lambda ,\mu \right)=\frac{1}{2\sigma^{2}}\sum_{i=1}^{M}{\left(d_{i}-g_{i}-\delta_{o}\right)}^{2}+\sum_{i=1}^{M}\lambda_{i}\left(g_{i}^{2}-{\left\|u-s_{i}^{\circ }\right\|}^{2}\right)+\sum_{i=1}^{M}\mu_{i}\left(g_{i}-\alpha_{i}\cdot y_{i}^{2}\right),$$

where $g={\left[g_{1},g_{2},\ldots ,g_{M}\right]}^{T}$ and $y={\left[y_{1},y_{2},\ldots ,y_{M}\right]}^{T}$. The Lagrange multipliers are denoted by $\lambda_{i}$ and $\mu_{i}$. Then, collecting these multipliers yields the vectors $\lambda ={\left[\lambda_{1},\lambda_{2},\ldots ,\lambda_{M}\right]}^{T}$ and $\mu ={\left[\mu_{1},\mu_{2},\ldots ,\mu_{M}\right]}^{T}$.

However, the Lagrangian function in (10) is not stable enough to miss the sufficient condition of strict local convexity, which is also confirmed in some preliminary works [22,23]. Following the analysis in [16], an augmented term is included to improve the convexity and stability of the Lagrangian function as:

(11) $$A\left(u,g,y\right)=\frac{C_{0}}{2}\left[\sum_{i=1}^{M}{\left(g_{i}^{2}-{\left\|u-s_{i}^{\circ }\right\|}^{2}\right)}^{2}+\sum_{i=1}^{M}{\left(g_{i}-\alpha_{i}\cdot y_{i}^{2}\right)}^{2}\right],$$

where $C_{0}$ is a positive constant. Therefore, the formula in (10) is modified as:

(12) $$L_{a}\left(u,g,\delta_{o},y,\lambda ,\mu \right)=\frac{1}{2\sigma^{2}}\sum_{i=1}^{M}{\left(d_{i}-g_{i}-\delta_{o}\right)}^{2}+\sum_{i=1}^{M}\lambda_{i}\left(g_{i}^{2}-{\left\|u-s_{i}^{\circ }\right\|}^{2}\right)+\sum_{i=1}^{M}\mu_{i}\left(g_{i}-\alpha_{i}\cdot y_{i}^{2}\right)\;+\frac{C_{0}}{2}\left[\sum_{i=1}^{M}{\left(g_{i}^{2}-{\left\|u-s_{i}^{\circ }\right\|}^{2}\right)}^{2}+\sum_{i=1}^{M}{\left(g_{i}-\alpha_{i}\cdot y_{i}^{2}\right)}^{2}\right]$$

It is worth pointing that at an equilibrium point $\left(u^{*},g^{*},\delta_{o}^{*},y^{*},\lambda^{*},\mu^{*}\right)$, the constraints in (9) are satisfied, i.e., ${\left(g_{i}^{*}\right)}^{2}-{\left\|u^{*}-s_{i}^{\circ }\right\|}^{2}=0$ and $g_{i}^{*}-\alpha_{i}\cdot {\left(y_{i}^{*}\right)}^{2}=0$, ∀i=1,2,…,M. Hence, the augmented term $A(u^{*},g^{*},y^{*})=0$. In other words, the original value of the objective function at minimum point will not be changed by the augmented term. Meanwhile, by introducing the augmented term, it will convexify the original problem and it is also helpful for accelerating the convergence of the neural networks [10]. Further discussion about the effectiveness of the augmented term will be analyzed in Section 5.

Applying (3) to the proposed Lagrangian function (12), the dynamics of the primal variable neurons are given by:

(13) $$\frac{du}{dt}=-\frac{\partial L_{a}}{\partial u}=2C_{0}\sum_{i=1}^{M}\left(g_{i}^{2}-{\left\|u-s_{i}^{\circ }\right\|}^{2}\right)\left(u-s_{i}^{\circ }\right)+2\sum_{i=1}^{M}\lambda_{i}\left(u-s_{i}^{\circ }\right),$$

(14) $$\frac{dg_{i}}{dt}=-\frac{\partial L_{a}}{\partial g_{i}}=\frac{1}{\sigma^{2}}\left(d_{i}-g_{i}-\delta_{o}\right)-2C_{0}\cdot g_{i}\left(g_{i}^{2}-{\left\|u-s_{i}^{\circ }\right\|}^{2}\right)-C_{0}\left(g_{i}-\alpha_{i}\cdot y_{i}^{2}\right)-2\lambda_{i}\cdot g_{i}-\mu_{i},$$

(15) $$\frac{d\delta_{o}}{dt}=-\frac{\partial L_{a}}{\partial \delta_{o}}=\frac{1}{\sigma^{2}}\sum_{i=1}^{M}\left(d_{i}-g_{i}-\delta_{o}\right),$$

(16) $$\frac{dy_{i}}{dt}=-\frac{\partial L_{a}}{\partial y_{i}}=2C_{0}\left(g_{i}-\alpha_{i}\cdot y_{i}^{2}\right)\alpha_{i}\cdot y_{i}+2\mu_{i}\cdot \alpha_{i}\cdot y_{i}.$$

Similarly, the dynamics of the Lagrangian neurons are:

(17) $$\frac{d\lambda_{i}}{dt}=\frac{\partial L_{a}}{\partial \lambda_{i}}=g_{i}^{2}-{\left\|u-s_{i}^{\circ }\right\|}^{2},$$

(18) $$\frac{d\mu_{i}}{dt}=\frac{\partial L_{a}}{\partial \mu_{i}}=g_{i}-\alpha_{i}\cdot y_{i}^{2}.$$

In the proposed LPNN method, the transient behavior of the network is governed by the differential equations defined in (13)–(18). Moreover, these differential equations contain two types of neurons, i.e., variable neurons and Lagrangian neurons. There are (2M+3) variable neurons for holding u, $g_{i}$, $y_{i}$, (i=1,2,…,M), and $\delta_{o}$, respectively. Besides, there are 2M Lagrangian neurons to hold $\lambda_{i}$ and $\mu_{i}$.

**Figure 1:**
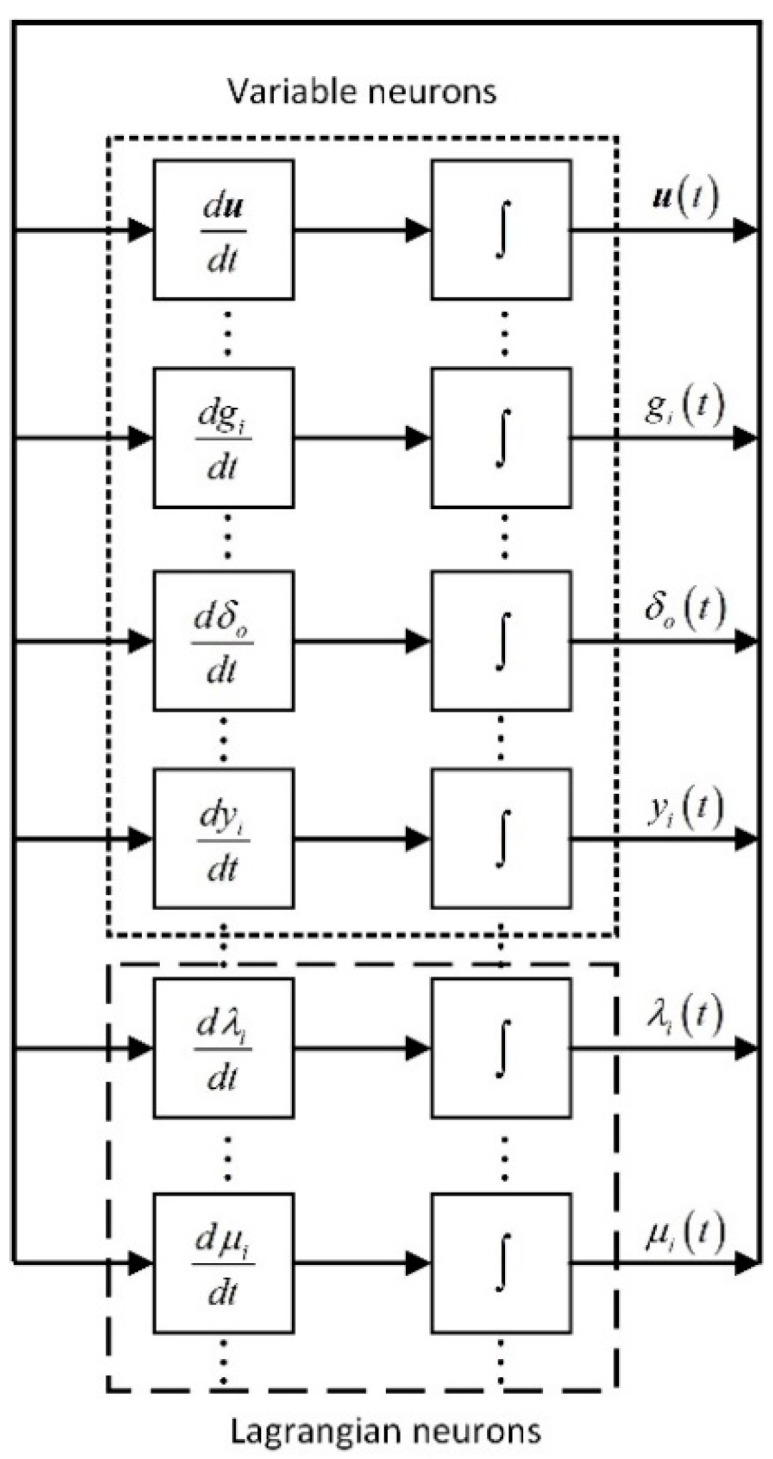
The structure of the proposed LPNN with unknown $t_{o}$.

Figure 1 illustrates the realization of the TOA-based LPNN model when the start transmission time is unknown. It is composed of (4M+2) function blocks and integrators. The inputs of the neurons are calculated by the function blocks, while the states of the neurons are updated by the integrators and then fed back to the function blocks. According to [24,25], the implementation procedure for LPNN is given in the Algorithm 1 below:

**Algorithm 1.** The TOA-Based LPNN Model with Unknown Start Transmission Time1. Based on the dynamic Equations (13)–(18), a neural network is built up at the beginning. 2. Set the initial values for $u,g,\delta_{o},y,\lambda ,\mu$ and incorporate the TOA measurements. Then, set the integration time t=0 and switch on the network. 3. The time derivative is first computed at each neuron and then passed to an integrator, which outputs the neuron state. 4. Feedback the output neuron states as the input of the neural network. 5. Repeat step 3 and step 4 until the network eventually reach an equilibrium point. 6. When the states of the neural network settle down, the network output states are the final estimations.

## 4. Extension to the Case with Inaccurate Sensor Positions

In the preceding source localization algorithm using TOA measurements, the sensor positions are assumed to be accurate. However, in some applications, the sensor positions are not known exactly. For example, the location bias may arise in the positioning system used in the sensor network, which can cause sensor position errors. Therefore, the data fusion center can only obtain the erroneous information of the sensor positions. From [14], the inexact information of the sensor coordinates can dramatically deteriorate the localization performance. To achieve reliable target localization, we need to take account of the impact of these position uncertainties.

Following the model of [14,26], the observed sensor positions are expressed as:

(19) $$s_{i}=\;s_{i}^{\circ }+\;\Delta s_{i},\;i=1,2,\ldots ,M,$$

where $\Delta s_{i}$ is sensor location error, which is assumed to be zero-mean Gaussian distributed with covariance matrix $E(\Delta s_{i}\cdot \Delta s_{i}^{T})=\sigma_{s,i}^{2}\cdot I_{2}=\sigma_{s}^{2}\cdot I_{2}$, where $I_{2}$ is the second-order identity matrix. Moreover, for simplicity, the sensor location error is also assumed to be independent of the measurement noise. Under these assumptions, the measurement data d and $s_{1},s_{2},\ldots ,s_{M}$ follow a joint Gaussian distribution and their joint PDF is represented as:

(20) $$p\left(d_{1},d_{2},\ldots ,d_{M},s_{1},s_{2},\ldots ,s_{M}|u,s_{1}^{\circ },s_{2}^{\circ },\ldots ,s_{M}^{\circ },\delta_{o}\right)\;={\left(2\pi \sigma^{2}\right)}^{-\frac{M}{2}}\exp\left(-\frac{1}{2\sigma^{2}}\sum_{i=1}^{M}{\left(d_{i}-\left\|u-s_{i}^{\circ }\right\|-\delta_{o}\right)}^{2}\right)\times {\left(2\pi \sigma_{s}^{2}\right)}^{-\frac{M}{2}}\exp(-\frac{1}{2\sigma_{s}^{2}}\sum_{i=1}^{M}{\left\|s_{i}-s_{i}^{\circ }\right\|}^{2}).$$

Define $\theta ={\left[u^{T},s_{1}^{T\circ },s_{2}^{T\circ },\ldots ,s_{M}^{T\circ },\delta_{o}\right]}^{T}$. The maximum log-likelihood estimation of θ is given by

(21) $$\hat{\theta }=\begin{array}{cc} \arg & \underset{\theta }{\max}\left(-\frac{1}{2\sigma^{2}}\sum_{i=1}^{M}{\left(d_{i}-\left\|u-s_{i}^{\circ }\right\|-\delta_{o}\right)}^{2}-\frac{1}{2\sigma_{s}^{2}}\sum_{i=1}^{M}{\left\|s_{i}-s_{i}^{\circ }\right\|}^{2}\right) \end{array}.$$

Similar to the reformulation for (7), (21) can be recast as:

(22) $$\begin{array}{l} \underset{\theta }{\min}\left(\frac{1}{2\sigma^{2}}\sum_{i=1}^{M}{\left(d_{i}-g_{i}-\delta_{o}\right)}^{2}+\frac{1}{2\sigma_{s}^{2}}\sum_{i=1}^{M}{\left\|s_{i}-s_{i}^{\circ }\right\|}^{2}\right)\\ s.t.\;g_{i}^{2}={\left\|u-s_{i}^{\circ }\right\|}^{2}\;,\\ \;g_{i}=\alpha_{i}\cdot y_{i}^{2},i=1,2,\ldots ,M \end{array}$$

where we have also introduced the additional variables $y_{i},i=1,2,\ldots ,M$ to transform the inequality constraints into equalities. Furthermore, we include the augmented term A(u,g,y) defined in (11) to convexify the objective function and improve the stability of the network. Consequently, the newly built Lagrangian function is given by:

(23) $$L_{a}\left(\theta ,g,y,\lambda ,\mu \right)=\frac{1}{2\sigma^{2}}\sum_{i=1}^{M}{\left(d_{i}-g_{i}-\delta_{o}\right)}^{2}+\frac{1}{2\sigma_{s}^{2}}\sum_{i=1}^{M}{\left\|s_{i}-s_{i}^{\circ }\right\|}^{2}+\sum_{i=1}^{M}\lambda_{i}\left(g_{i}^{2}-{\left\|u-s_{i}^{\circ }\right\|}^{2}\right)+\sum_{i=1}^{M}\mu_{i}\left(g_{i}-\alpha_{i}\cdot y_{i}^{2}\right)\;+\frac{C_{0}}{2}\left[\sum_{i=1}^{M}{\left(g_{i}^{2}-{\left\|u-s_{i}^{\circ }\right\|}^{2}\right)}^{2}+\sum_{i=1}^{M}{\left(g_{i}-\alpha_{i}\cdot y_{i}^{2}\right)}^{2}\right]$$

We notice that there are additional 2M variable neurons for holding $s_{1}^{\circ },s_{2}^{\circ },\ldots ,s_{M}^{\circ }$ compared with (12). Besides, the dynamics of u, $g_{i}$, $y_{i}$, $\delta_{o}$, $\lambda_{i}$ and $\mu_{i}$ are the same as those in (13)–(18). For the states of unknown sensor positions, their dynamics of the network are given by:

(24) $$\frac{ds_{i}^{\circ }}{dt}=-\frac{\partial L_{a}}{\partial s_{i}^{\circ }}=\frac{1}{\sigma_{s}^{2}}\left(s_{i}-s_{i}^{\circ }\right)-2C_{0}\left(g_{i}^{2}-{\left\|u-s_{i}^{\circ }\right\|}^{2}\right)\left(u-s_{i}^{\circ }\right)-2\lambda_{i}\left(u-s_{i}^{\circ }\right)$$

**Figure 2:**
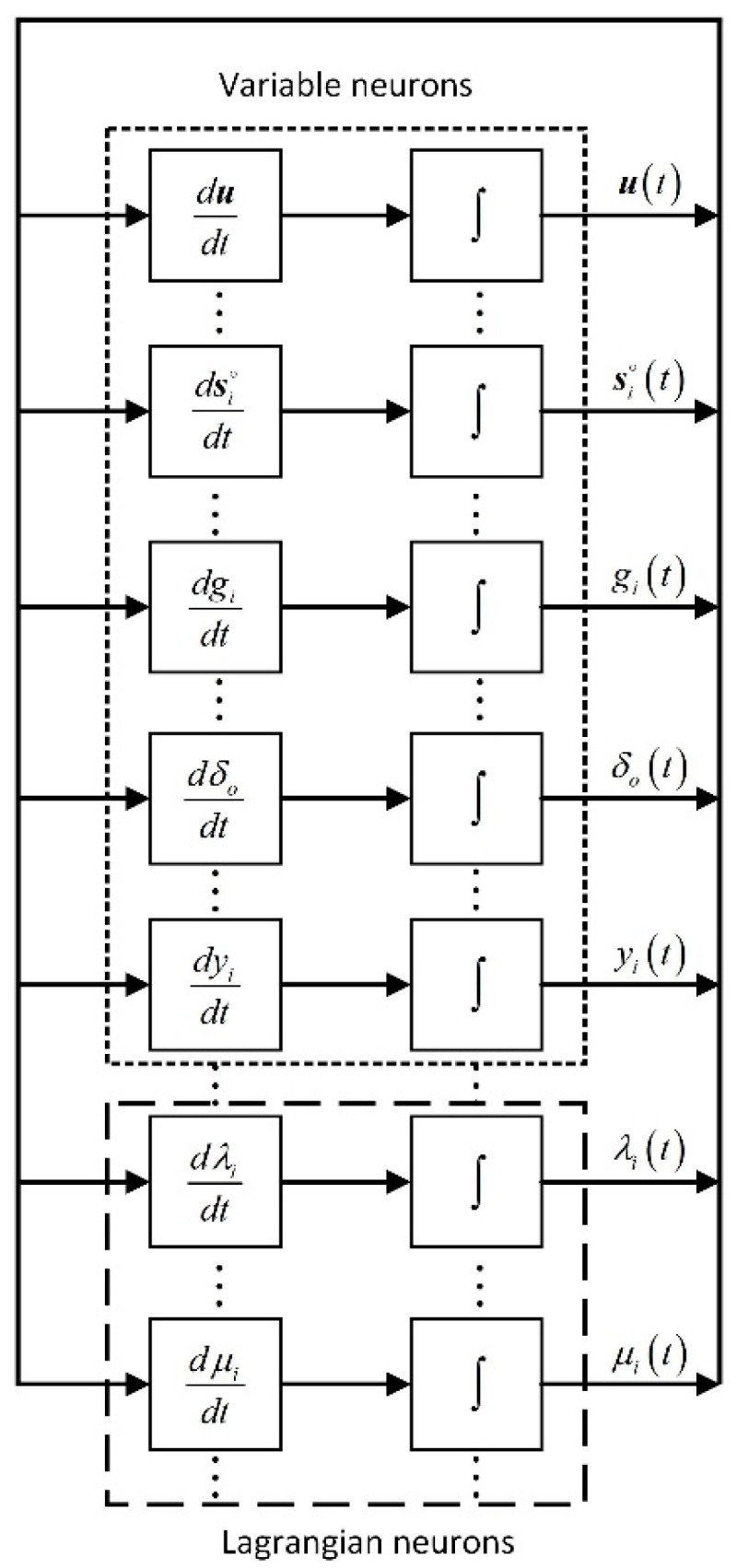
The structure of the proposed LPNN with unknown start transmission time and sensor position uncertainties.

The realization of the TOA-based LPNN model considering the unknown start transmission time when the sensor positions are not accurately known is illustrated in Figure 2. It is composed of (5M+2) function blocks and integrators, and their functions are the same as stated in Section 3. Moreover, it is worth noting that comparing the network developed in the previous section with that defined in this section, there is an interchangeable structure between these two kinds of networks. In hardware realization, the network for the case with sensor position uncertainties can be implemented by adding extra neurons representing additional variables, such as $s_{i}^{\circ }$, i=1,2,…,M, to the network for the original problem formulated in Section 3.

The implementation procedure for LPNN is given in the Algorithm 2 below.

**Algorithm 2.** The TOA-Based LPNN Model with Unknown Start Transmission Time and Sensor Position Uncertainties1. Based on the dynamic Equations (13)–(18) and (24), a neural network is built up at the beginning. 2. Set the initial values for θ,g,y,λ,μ and incorporate the TOA measurements. Then, set the integration time t=0 and switch on the network. 3. The time derivative is first computed at each neuron and then passed to an integrator, which outputs the neuron state. 4. Feedback the output neuron states as the input of the neural network. 5. Repeat the step 3 and step 4 until the network eventually reaches an equilibrium point. 6. When the states of the neural network settle down, the network output states are the final estimations.

In this paper, the implementation procedure for LPNN is simulated by utilizing the Runge-Kutte method, which is embedded in MATLAB ode solver, to solve the differential equations. Besides, when utilizing neural network for optimization, it is necessary to guarantee the convergence and stability of the network, which will be examined in the next section.

## 5. The Convergence and Stability Analysis

Considering the existence of clock asynchronization and sensor position uncertainties, the convergence and stability of the two neural networks are investigated in this section. These two properties are then illustrated by numerical experiments using both the noise-free and noisy TOA measurements.

### 5.1. The LPNN Model with Clock Asynchronization

According to [16], the saddle point property is analyzed to illustrate how the neural network is able to find the optimal solution. As stated before, the variable neurons decrease the Lagrangian function while the Lagrangian neurons constrain the variables within the feasible region during the dynamic process of the neural network. Specifically, by taking time derivation of Lagrangian function (2) when λ and x are kept constant respectively, and combining the dynamics of the network in (3), we have the:

(25) $$\begin{array}{l} {\frac{dL\left(x,\lambda \right)}{dt}|}_{\lambda =\mathrm{constant}}=\sum_{i=1}^{n}\frac{\partial L\left(x,\lambda \right)}{\partial x_{i}}\frac{dx_{i}}{dt}=-\sum_{i=1}^{n}{\left(\frac{dx_{i}}{dt}\right)}^{2}\leq 0,\\ {\frac{dL\left(x,\lambda \right)}{dt}|}_{x=\mathrm{constant}}=\sum_{i=1}^{m}\frac{\partial L\left(x,\lambda \right)}{\partial \lambda_{i}}\frac{d\lambda_{i}}{dt}=\sum_{i=1}^{n}{\left(\frac{d\lambda_{i}}{dt}\right)}^{2}\geq 0. \end{array}\;$$

Consequently, Equation (25) indicates that, from an initial point, the Lagrangian function is decreased by x and increased by λ along the searching process of the neural network, until the network reaches an equilibrium at which:

(26) $${\left(dL/dt\right)|}_{\left(x^{*},\lambda^{*}\right)}={\left(\partial L/\partial x^{T}\right)\left(dx/dt\right)|}_{\left(x^{*},\lambda^{*}\right)}+{\left(\partial L/\partial \lambda^{T}\right)\left(d\lambda /dt\right)|}_{\left(x^{*},\lambda^{*}\right)}=0.$$

Thus $\left(x^{*},\lambda^{*}\right)$ is a saddle point of L(x,λ), which satisfies:

(27) $$L\left(x^{*},\lambda \right)\leq L\left(x^{*},\lambda^{*}\right)\leq L\left(x,\lambda^{*}\right).$$

The saddle point property is a sufficient condition for optimality [16] and reference therein. In addition, it is further required to be asymptotically stable for a network to be of practical sense, which guarantees that the network can always converge to an equilibrium point from any initial state within the attraction domain.

Let $x^{*}$ be a local minimum point of f(x), and $\lambda^{*}$ be the corresponding Lagrange multiplier, thus $\left(x^{*},\lambda^{*}\right)$ is an equilibrium point of L(x,λ). As proved in [16], if the Hessian matrix of the Lagrangian function at $\left(x^{*},\lambda^{*}\right)$ is positive definite and the gradient vectors of the constraints are linearly independent, then $\left(x^{*},\lambda^{*}\right)$ is an asymptotically stable point of the network.

We first prove that in our proposed LPNN, the gradient vectors of the constraints are linearly independent at the equilibrium point $\left(x^{*},\lambda^{*},\mu^{*}\right)$. Here, the optimization variable vector x and the equality constraints h are denoted by:

(28) $$x={\left[u^{T},g^{T},y^{T},\delta_{o}\right]}^{T},$$

(29) $$h_{i}\left(x\right)=h_{i}\left(u,g,y,\delta_{o}\right)=g_{i}^{2}-{\left\|u-s_{i}^{\circ }\right\|}^{2},$$

(30) $$h_{M+i}\left(x\right)=h_{M+i}\left(u,g,y,\delta_{o}\right)=g_{i}-\alpha_{i}\cdot y_{i}^{2},\;i=1,2,\ldots ,M,$$

Thus, the gradient vectors $\left\{\nabla_{x}h_{1}(x^{*}),\ldots ,\nabla_{x}h_{2M}(x^{*})\right\}$ at an equilibrium point $x^{*}$ can be computed as:

(31) $${\frac{\partial h_{i}\left(x\right)}{\partial x}|}_{x=x^{*}}={\left[\begin{array}{cccccc} -2{\left(u^{*}-s_{i}^{\circ }\right)}^{T} & 0_{1\times (i-1)} & 2g_{i}^{*} & 0_{1\times (M-i)} & 0_{1\times M} & 0 \end{array}\right]}^{T},$$

(32) $${\frac{\partial h_{M+i}\left(x\right)}{\partial x}|}_{x=x^{*}}={\left[\begin{array}{cccccccc} 0_{1\times 2} & 0_{1\times (i-1)} & 1 & 0_{1\times (M-i)} & 0_{1\times (i-1)} & -2\alpha_{i}\cdot y_{i}^{*} & 0_{1\times (M-i)} & 0 \end{array}\right]}^{T}.$$

Then, from (31) and (32), putting all gradient vectors together forms (33) given by:

(33) $$\left\{\left[\begin{array}{c} -2u^{*}+2s_{1}^{\circ }\\ 2g_{1}^{*}\\ 0_{(M-2)\times 1}\\ 0\\ 0\\ 0_{(M-2)\times 1}\\ 0\\ 0 \end{array}\right],\ldots ,\left[\begin{array}{c} -2u^{*}+2s_{M}^{\circ }\\ 0\\ 0_{(M-2)\times 1}\\ 2g_{M}^{*}\\ 0\\ 0_{(M-2)\times 1}\\ 0\\ 0 \end{array}\right],\left[\begin{array}{c} 0_{2\times 1}\\ 1\\ 0_{(M-2)\times 1}\\ 0\\ -2\alpha_{1}\cdot y_{1}^{*}\\ 0_{(M-2)\times 1}\\ 0\\ 0 \end{array}\right],\ldots ,\left[\begin{array}{c} 0_{2\times 1}\\ 0\\ 0_{(M-2)\times 1}\\ 1\\ 0\\ 0_{(M-2)\times 1}\\ -2\alpha_{M}\cdot y_{M}^{*}\\ 0 \end{array}\right]\right\}.$$

From (33), it can be found that the gradient vectors are linearly independent as long as $g_{i}^{*}\neq 0$ and $u^{*}-s_{i}^{\circ }\neq 0$ for all i=1,2,…,M. That is, the estimated source position should not coincide with any sensor positions. This can be guaranteed in most of the common source localization conditions where the sensor positions are different from that of the source.

Then we show that the Hessian matrix of the proposed Lagrangian function is positive definite at the equilibrium point $\left(x^{*},\lambda^{*},\mu^{*}\right)$ by adding the augmented term. Based on the formulations in (10), (12) and (28)–(30), the augmented Lagrangian function is formulated as:

(34) $$L_{a}\left(x,\lambda ,\mu \right)=L_{c}\left(x,\lambda ,\mu \right)+\frac{C_{0}}{2}{\left\|h\left(x\right)\right\|}^{2}.$$

We can directly obtain the Hessian matrix of our augmented Lagrangian function at the equilibrium point as:

(35) $$\nabla_{xx}^{2}L_{a}\left(x^{*},\lambda^{*},\mu^{*}\right)=\nabla_{xx}^{2}L_{c}\left(x^{*},\lambda^{*},\mu^{*}\right)+C_{0}\nabla h\left(x^{*}\right)\nabla h{\left(x^{*}\right)}^{T},$$

where the Hessian matrix of $L_{c}(x,\lambda ,\mu )$ is denoted by $\nabla_{xx}^{2}L_{c}(x^{*},\lambda^{*},\mu^{*})$. With the aid of augmented term, Equation (35) indicates that the local convexity assumption $\nabla_{xx}^{2}L_{a}(x^{*},\lambda^{*},\mu^{*})>0$ can be satisfied when $C_{0}$ is taken sufficient large. Hence, $\left(x^{*},\lambda^{*},\mu^{*}\right)$ is an asymptotically stable point of the proposed neural network.

**Figure 3:**
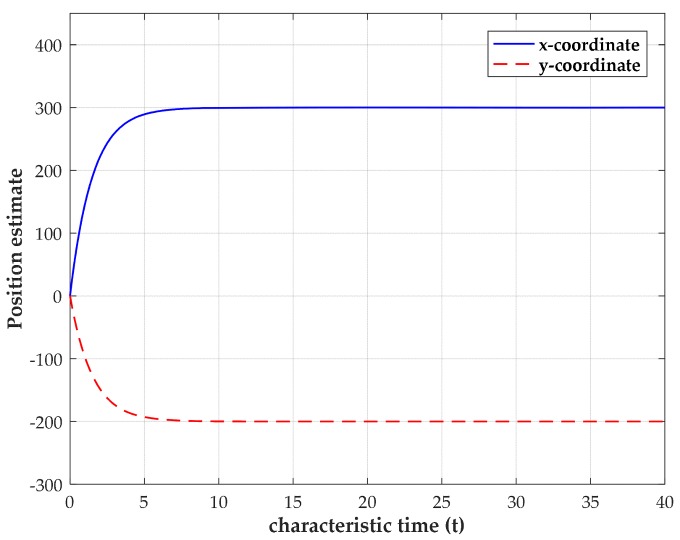
Transient behavior of the estimated source coordinates with the source position located at (300, −200) m.

To obtain more insight, we examine the convergence and stability of our method using the noise-free and noisy TOA measurements by simulations. In these numerical experiments, four sensors are deployed at (2000, 2000) m, (2000, −2000) m, (−2000, 2000) m and (−2000, −2000) m, while the source is located at (300, −200) m. The initial value of the variables are randomly chosen between 0 and 1. The constants $C_{0}$ and $\alpha_{i}$ are chosen as $C_{0}=\;5$ and $\alpha_{i}=\;10$ for all i=1,2,…,M in simulations. When the TOA measurements are noise-free, the transient behavior of the estimated source position for a single trial using four sensors is shown in Figure 3. It can be observed that the output states of network can finally converge to the true source coordinates after a short time of calculation. When it converges, the neural network can maintain the stable outputs as the characteristic time (characteristic time is used in MATLAB ode solver to specify the interval of integration) increases. The result of the noise-free case indicates that the convergence of the network is an intrinsic propertyof our model.

**Figure 4:**
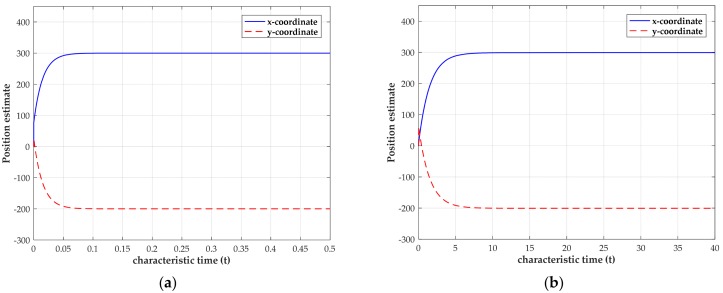
Transient behavior of the estimated source coordinates with the source position located at (300, −200) m. (**a**) Transient behavior when $\sigma^{2}=0.01\;m^{2}$; (**b**) transient behavior when $\sigma^{2}=1\;m^{2}$.

In practical, the obtained TOAs are often contaminated by measurement noise. When the network is tested with the noisy TOA measurements under two measurement noise levels, the transient behaviors of the estimated source coordinates using four sensors are shown in Figure 4. Due to the clock offset, the range deviation $\delta_{o}$ is randomly generated from the zero mean and variance of $400\;m^{2}$ Gaussian distribution in our simulations, though it is actually not a random variable. Thus, we could cover a much wider range of deviation to examine our method by performing this operation. In Figure 4, for small noise level, $\sigma^{2}=0.01\;m^{2}$, the network can settle down within 0.1 characteristic time. When we increase the noise level to $\sigma^{2}=1\;m^{2}$, the network can still converge and settle down at an equilibrium point within 10 characteristic times. The difference of convergence speed further indicates that it requires more time for the network to settle down when the noise is intensive. Moreover, from Figure 4, we can also observe that the converged position estimate is close to (300, −200) m.

### 5.2. The LPNN Model with Clock Asynchronization and Sensor Position Uncertainties

The proof of the convergence and stability of the network is similar to that in Section 5.1. Since the Equations (25)–(27) have provided a general analysis of the convergence of LPNN, the stability of the network is mainly discussed here.

According to the sufficient conditions stated in Section 5.1, we first prove that the gradient vectors of the constraints are linearly independent at the equilibrium point $\left(x^{*},\lambda^{*},\mu^{*}\right)$ in this proposed LPNN. Here, the optimization variable vector x and the equality constraints h are denoted by:

(36) $$x={\left[u^{T},s_{1}^{T\circ },s_{2}^{\circ T},\ldots ,s_{M}^{\circ T},g^{T},y^{T},\delta_{o}\right]}^{T},$$

(37) $$h_{i}\left(x\right)=h_{i}\left(u,s_{1}^{\circ },s_{2}^{\circ },\ldots ,s_{M}^{\circ },g,y,\delta_{o}\right)=g_{i}^{2}-{\left\|u-s_{i}^{\circ }\right\|}^{2},$$

(38) $$h_{M+i}\left(x\right)=h_{M+i}\left(u,s_{1}^{\circ },s_{2}^{\circ },\ldots ,s_{M}^{\circ },g,y,\delta_{o}\right)=g_{i}-\alpha_{i}\cdot y_{i}^{2},i=1,2,\ldots ,M,$$

Thus, the gradient vectors $\left\{\nabla_{x}h_{1}(x^{*}),\ldots ,\nabla_{x}h_{2M}(x^{*})\right\}$ at an equilibrium point $x^{*}$ can be computed as:

(39) $${\frac{\partial h_{i}\left(x\right)}{\partial x}|}_{x=x^{*}}={\left[\begin{array}{ccccccccc} -2{\left(u^{*}-s_{i}^{\circ *}\right)}^{T} & 0_{1\times 2(i-1)} & 2{\left(u^{*}-s_{i}^{\circ *}\right)}^{T} & 0_{1\times 2(M-i)} & 0_{1\times 2(i-1)} & 2g_{i}^{*} & 0_{1\times (M-i)} & 0_{1\times M} & 0 \end{array}\right]}^{T},$$

(40) $${\frac{\partial h_{M+i}\left(x\right)}{\partial x}|}_{x=x^{*}}={\left[\begin{array}{ccccccccc} 0_{1\times 2} & 0_{1\times 2M} & 0_{1\times (i-1)} & 1 & 0_{1\times (M-i)} & 0_{1\times (i-1)} & -2\alpha_{i}\cdot y_{i}^{*} & 0_{1\times (M-i)} & 0 \end{array}\right]}^{T}.$$

Then, from (39) and (40), collecting all gradient vectors together forms (41) expressed by:

(41) $$\{\left[\begin{array}{c} -2u^{*}+2s_{1}^{\circ *}\\ 2u^{*}-2s_{1}^{\circ *}\\ 0_{2(M-1)\times 1}\\ 2g_{1}^{*}\\ 0_{(M-2)\times 1}\\ 0\\ 0_{(M-2)\times 1}\\ 0\\ 0 \end{array}\right],\ldots ,\left[\begin{array}{c} -2u^{*}+2s_{M}^{\circ *}\\ 0_{2(M-1)\times 1}\\ 2u^{*}-2s_{M}^{\circ *}\\ 0\\ 0_{(M-2)\times 1}\\ 2g_{M}^{*}\\ 0_{(M-2)\times 1}\\ 0\\ 0 \end{array}\right],\left[\begin{array}{c} 0_{2\times 1}\\ 0_{2M\times 1}\\ 1\\ 0_{(M-2)\times 1}\\ 0\\ -2\alpha_{1}\cdot y_{1}^{*}\\ 0_{(M-2)\times 1}\\ 0\\ 0 \end{array}\right],\ldots ,\left[\begin{array}{c} 0_{2\times 1}\\ 0_{2M\times 1}\\ 0\\ 0_{(M-2)\times 1}\\ 1\\ 0\\ 0_{(M-2)\times 1}\\ -2\alpha_{M}\cdot y_{M}^{*}\\ 0 \end{array}\right]\}.$$

From (41), it can be found that the gradient vectors are linearly independent, as long as the estimated source position is different from one of the sensor positions.

It should also be noticed that, when $C_{0}$ is sufficiently large, the Hessian matrix of the proposed Lagrangian function is positive definite at equilibrium point $\left(x^{*},\lambda^{*},\mu^{*}\right)$ by adding the augmented term according to the proof provided by Equations (34) and (35). Therefore, the asymptotically stable property of the proposed neural network can be guaranteed.

**Figure 5:**
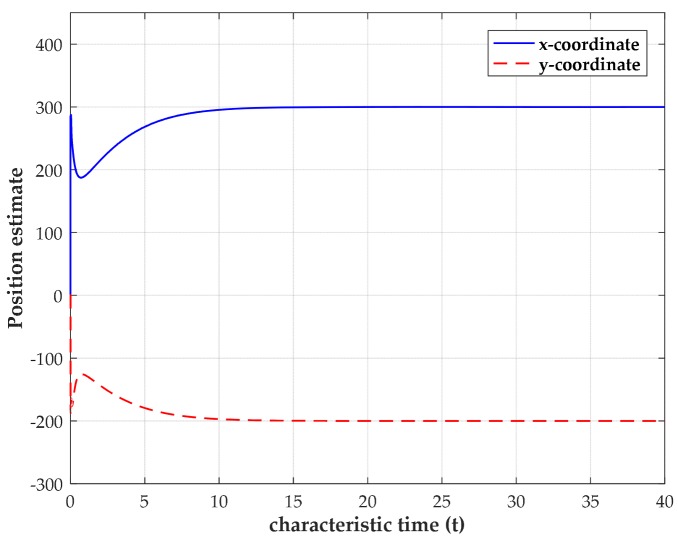
Transient behavior of the estimated source coordinates using noise-free TOA measurements with source position located at (300, −200) m.

The convergence and stability of this LPNN model is also illustrated by the numerical experiment utilizing noise-free and noisy TOA measurements, respectively. The simulation settings are the same as in Section 5.1. The transient behavior of the estimated source position for a single trial using noise-free TOA measurements is firstly shown in Figure 5. Figure 5 exhibits that, after a short time of oscillation, the output states of network can eventually converge to the true source coordinates. The neural network can maintain the stable outputs as the characteristic time increases when it converges.

**Figure 6:**
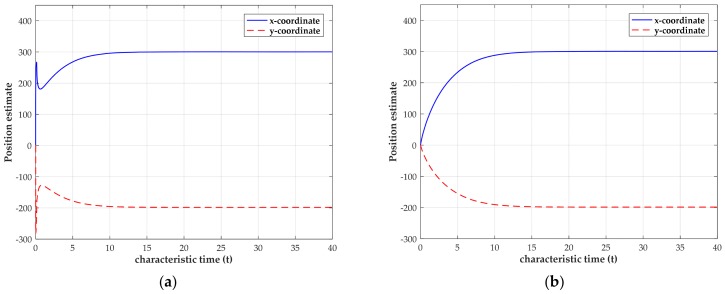
Transient behavior of the estimated source coordinates using noisy TOA measurements with the source position located at (300, −200) m. (**a**) Transient behavior when $\sigma^{2}=0.01\;m^{2}$; (**b**) transient behavior when $\sigma^{2}=1\;m^{2}$.

The transient behaviors of the estimated source position under two measurement noise levels are shown in Figure 6, when the sensor positions are not known exactly. The simulation settings are the same as mentioned above. Additionally, the sensor location error variance $\sigma_{s}^{2}$ is set as 1 m^2^ in the simulation. Figure 6 shows that, for small noise level $\sigma^{2}=0.01{\;m}^{2}$, the network can settle down in a shorter time than the case with a large noise level, which verifies that the noise level can affect the convergence speed again. Besides, in both the cases, the network can approximately converge to the true source position.

## 6. The Derivation of CRLB

CRLB is usually seen as a benchmark of the highest accuracy that any unbiased estimator can achieve. In this section, the CRLB with clock asynchronization using TOA measurements and the CRLB with both clock asynchronization and sensor position uncertainties are derived explicitly.

### 6.1. The CRLB with Clock Asynchronization

In this section, we first derive CRLB for the TOA-based localization problem without clock synchronization in Section 3 based on the relevant work of [11]. According to the PDF of the range measurements in (6), the log-likelihood function is given by:

(42) $$L_{o}\left(u,\delta_{o}\right)=-\frac{1}{2\sigma^{2}}\sum_{i=1}^{M}{\left(d_{i}-\left\|u-s_{i}^{\circ }\right\|-\delta_{o}\right)}^{2}.$$

where the constant term has been ignored. Hence, the fisher information matrix (FIM) is defined as follows:

(43) $$F_{1}=-\left[\begin{array}{cc} E\left\{\frac{\partial^{2}L_{o}}{\partial u\partial u^{T}}\right\} & E\left\{\frac{\partial^{2}L_{o}}{\partial u\partial \delta_{o}}\right\}\\ E\left\{\frac{\partial^{2}L_{o}}{\partial \delta_{o}\partial u^{T}}\right\} & E\left\{\frac{\partial^{2}L_{o}}{\partial \delta_{o}^{2}}\right\} \end{array}\right],$$

where the submatrices of $F_{1}\in {\mathbb{R}}^{3\times 3}$ are computed as:

(44) $$E\left\{\frac{\partial^{2}L_{o}}{\partial u\partial u^{T}}\right\}=-\frac{1}{\sigma^{2}}\sum_{i=1}^{M}\frac{\left(u-s_{i}^{\circ }\right){\left(u-s_{i}^{\circ }\right)}^{T}}{{\left\|u-s_{i}^{\circ }\right\|}^{2}},$$

(45) $$E\left\{\frac{\partial^{2}L_{o}}{\partial u\partial \delta_{o}}\right\}=-\frac{1}{\sigma^{2}}\sum_{i=1}^{M}\frac{\left(u-s_{i}^{\circ }\right)}{\left\|u-s_{i}^{\circ }\right\|},$$

(46) $$E\left\{\frac{\partial^{2}L_{o}}{\partial \delta_{o}\partial u^{T}}\right\}=-\frac{1}{\sigma^{2}}\sum_{i=1}^{M}\frac{{\left(u-s_{i}^{\circ }\right)}^{T}}{\left\|u-s_{i}^{\circ }\right\|},$$

(47) $$E\left\{\frac{\partial^{2}L_{o}}{\partial \delta_{o}^{2}}\right\}=-\frac{M}{\sigma^{2}}.$$

Therefore, the CRLB for source localization with clock asynchronization is given by the sum of the first two diagonal elements of $F_{1}^{-1}$.

### 6.2. The CRLB with Clock Asynchronization and Sensor Position Uncertainties

In this section, the case with both clock asynchronization and inaccurate sensor positions is considered. According to (20) and (21), the log-likelihood function is expressed as:

(48) $$L_{o}\left(\theta \right)=-\frac{1}{2\sigma^{2}}\sum_{i=1}^{M}{\left(d_{i}-\left\|u-s_{i}^{\circ }\right\|-\delta_{o}\right)}^{2}-\frac{1}{2\sigma_{s}^{2}}\sum_{i=1}^{M}{\left\|s_{i}-s_{i}^{\circ }\right\|}^{2}.$$

Furthermore, the FIM $F_{2}\in {\mathbb{R}}^{\left(2M+3\right)\times \left(2M+3\right)}$ in this case is defined as:

(49) $$F_{2}=-\left[\begin{array}{ccccc} E\left\{\frac{\partial^{2}L_{o}\left(\theta \right)}{\partial u\partial u^{T}}\right\} & E\left\{\frac{\partial^{2}L_{o}\left(\theta \right)}{\partial u\partial s_{1}^{\circ T}}\right\} & \cdots & E\left\{\frac{\partial^{2}L_{o}\left(\theta \right)}{\partial u\partial s_{M}^{\circ \;T}}\right\} & E\left\{\frac{\partial^{2}L_{o}\left(\theta \right)}{\partial u\partial \delta_{o}}\right\}\\ E\left\{\frac{\partial^{2}L_{o}\left(\theta \right)}{\partial s_{1}^{\circ }\partial u^{T}}\right\} & E\left\{\frac{\partial^{2}L_{o}\left(\theta \right)}{\partial s_{1}^{\circ }\partial s_{1}^{\circ T}}\right\} & \cdots & E\left\{\frac{\partial^{2}L_{o}\left(\theta \right)}{\partial s_{1}^{\circ }\partial s_{M}^{\circ T}}\right\} & E\left\{\frac{\partial^{2}L_{o}\left(\theta \right)}{\partial s_{1}^{\circ }\partial \delta_{o}}\right\}\\ \vdots & \vdots & \ddots & \vdots & \vdots \\ E\left\{\frac{\partial^{2}L_{o}\left(\theta \right)}{\partial s_{M}^{\circ }\partial u^{T}}\right\} & E\left\{\frac{\partial^{2}L_{o}\left(\theta \right)}{\partial s_{M}^{\circ }\partial {s_{1}^{\circ }}^{T}}\right\} & \cdots & E\left\{\frac{\partial^{2}L_{o}\left(\theta \right)}{\partial s_{M}^{\circ }\partial {s_{M}^{\circ }}^{T}}\right\} & E\left\{\frac{\partial^{2}L_{o}\left(\theta \right)}{\partial s_{M}^{\circ }\partial \delta_{o}}\right\}\\ E\left\{\frac{\partial^{2}L_{o}\left(\theta \right)}{\partial \delta_{o}\partial u^{T}}\right\} & E\left\{\frac{\partial^{2}L_{o}\left(\theta \right)}{\partial \delta_{o}\partial {s_{1}^{\circ }}^{T}}\right\} & \cdots & E\left\{\frac{\partial^{2}L_{o}\left(\theta \right)}{\partial \delta_{o}\partial {s_{M}^{\circ }}^{T}}\right\} & E\left\{\frac{\partial^{2}L_{o}\left(\theta \right)}{\partial \delta_{o}^{2}}\right\} \end{array}\right]$$

Note that in (49), $E\left\{\frac{\partial^{2}L_{o}\left(\theta \right)}{\partial u\partial u^{T}}\right\}$, $E\left\{\frac{\partial^{2}L_{o}\left(\theta \right)}{\partial u\partial \delta_{o}}\right\}$, $E\left\{\frac{\partial^{2}L_{o}\left(\theta \right)}{\partial \delta_{o}\partial u^{T}}\right\}$, and $E\left\{\frac{\partial^{2}L_{o}\left(\theta \right)}{\partial \delta_{o}^{2}}\right\}$ are equal to (44)–(47), respectively. Other submatrices can be directly computed by:

(50) $$E\left\{\frac{\partial^{2}L_{o}\left(\theta \right)}{\partial u\partial s_{i}^{\circ T}}\right\}=E\left\{\frac{\partial^{2}L_{o}\left(\theta \right)}{\partial s_{i}^{\circ }\partial u^{T}}\right\}=\frac{1}{\sigma^{2}}\frac{\left(u-s_{i}^{\circ }\right){\left(u-s_{i}^{\circ }\right)}^{T}}{{\left\|u-s_{i}^{\circ }\right\|}^{2}},$$

(51) $$E\left\{\frac{\partial^{2}L_{o}\left(\theta \right)}{\partial s_{i}^{\circ }\partial \delta_{o}}\right\}=E{\left\{\frac{\partial^{2}L_{o}\left(\theta \right)}{\partial \delta_{o}\partial s_{i}^{\circ T}}\right\}}^{T}=\frac{1}{\sigma^{2}}\frac{\left(u-s_{i}^{\circ }\right)}{\left\|u-s_{i}^{\circ }\right\|},$$

(52) $$E\left\{\frac{\partial^{2}L_{o}\left(\theta \right)}{\partial s_{i}^{\circ }\partial s_{j}^{\circ T}}\right\}=\{\begin{array}{ll} 0_{2\times 2} & ,\;i\neq j\\ \frac{1}{\sigma^{2}}\frac{\left(u-s_{i}^{\circ }\right){\left(u-s_{i}^{\circ }\right)}^{T}}{{\left\|u-s_{i}^{\circ }\right\|}^{2}}+\frac{1}{\sigma_{s}^{2}}I_{2\times 2} & ,i=j \end{array},\;i,j=1,\ldots ,M.$$

The CRLB for u in this case is the sum of the first two diagonal elements of $F_{2}^{-1}$.

## 7. Simulation Results

In this section, several simulations are conducted to evaluate the localization performance and robustness of the two LPNN-based target localization approaches developed in this paper. The localization algorithm developed in this paper is compared with the classic Taylor method [10], the two-stage WLS [13], and the corresponding CRLB derived in Section 6, as well as the ML method employed in [11], when the sensor positions are actually known.

From our preliminary formulation process, the dynamics of the neural networks is defined by a set of differential equations. Hence, LPNN dynamics are simulated by utilizing the MATLAB ode solver. The initial value of the variables is initialized as numbers randomly chosen between 0 and 1. Our intensive simulations show that it is sufficient enough to guarantee the convergence speed and stability of the proposed LPNN algorithm when $C_{0}\geq 5$ and there is little difference in localization performance in terms of the mean square error (MSE), given different $C_{0}\geq 5$. Thus, we choose $C_{0}=5$ in our simulation. Additionally, the value of $\alpha_{i}$ will only adjust the convergence speed and it has a minute impact on the ultimate estimate. Hence, any value that satisfies $\alpha_{i}\geq 1$ is appropriate. Here, we set $\alpha_{i}=10$ for all i=1,2,…,M.

**Figure 7:**
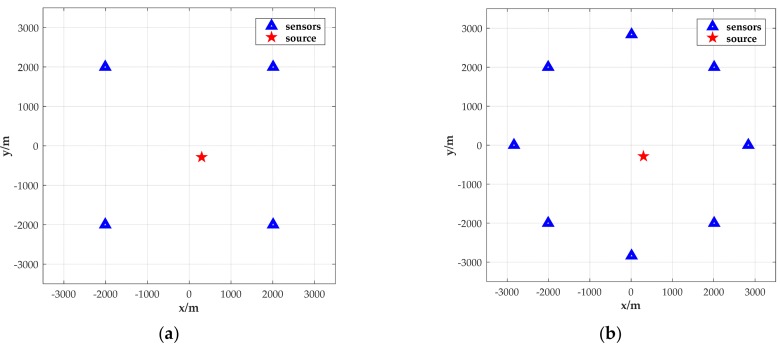
Positions of the sensors and source used in the simulation with the source position located at (300, −280) m. (**a**) Positions of four sensors and a source; (**b**) positions of eight sensors and a source.

In the first test, we consider only the time asynchronization case. Figure 7 shows the geometry where four sensors are located at (−2000, −2000) m, (−2000, 2000) m, (2000, −2000) m, and (2000, 2000) m, while the source is located at (300, −280) m. The geometry of eight sensors is also shown in Figure 7, which are located at (2000, −2000) m, $\left(0,\;-2000\sqrt{2}\right)$ m, (−2000, −2000) m, $\left(-2000\sqrt{2},\;0\right)$ m, (−2000, 2000) m, $\left(0,\;2000\sqrt{2}\right)$ m, (2000, 2000) m, and $\left(2000\sqrt{2},\;0\right)$ m, respectively.

**Figure 8:**
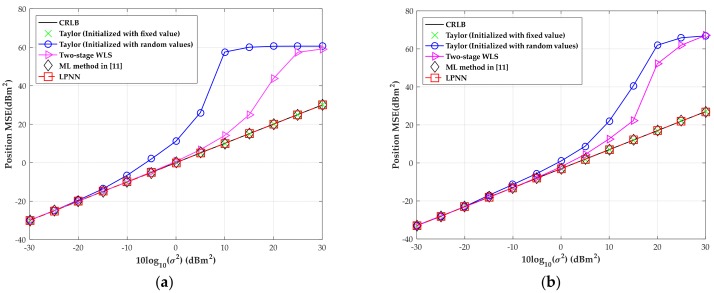
MSE versus level of measurement noise with the source position located at (300, −280) m. (**a**) Four sensors; (**b**) eight sensors.

The MSE versus the level of measurement noise with moderate time offset is shown in Figure 8 when the source is located at (300, −280) m. The range deviation variance, due to the clock offset, is fixed at 400 m^2^ and the MSEs are obtained through 1000 independent Monte-Carlo runs in our simulation. We also include the results of three numerical algorithms, namely the ML method employed in [11], the two-stage WLS and the classic Taylor algorithm initialized with fixed and random values, respectively, as well as the CRLB for comparison. Additionally, the initial source position is extracted within the center of the convex hull between the receiving sensors and the initial range deviation is set as zero for the Taylor method and the ML method in [11]. Under the same simulation settings, we then increase the number of sensors to eight. The corresponding MSEs versus the level of measurement noise with moderate time offset are also shown in Figure 8.

Figure 8 shows that the LPNN method can attain CRLB over a wide measurement noise condition as well as the ML method in [11] and the Taylor method initialized with a fixed initial values of source position and range deviation. However, once the Taylor method is badly initialized, namely the initial source position is extracted randomly within the convex hull between the sensors and the initial range deviation is also randomly extracted between (–100, 100) m, as shown in the figures, its MSE immediately deviates from the CRLB. However, this will not happen in our LPNN method and the ML method in [11]. The sensitivity to the initial value of the proposed method and the iterative Taylor method and ML method in [11] is examined in the fourth test. Moreover, even though the two-stage WLS algorithm has a higher noise threshold than the Taylor method, it will fail to give a reasonable estimate when the measurement noise is intensive. Besides, all the algorithms can obtain a promotion of estimate performance when we increase the number of sensors to eight. By comparison, the LPNN method developed in this article is still optimal as the ML method employed in [11].

It is worth pointing out that even though the ML method in [11] and proposed LPNN method have similar performance, these two methods are quite different. Starting from the same purpose of solving the positioning problem, the iterative ML is an efficient numerical method, while the LPNN framework is trying to establish a neural network circuit that can be implemented by analog neural circuit to solve the positioning problem. If the source location can be directly solved by an analog circuit without calculation on digital computers, it will attract applications with limited computing resources. Therefore, the LPNN framework provides a new method and idea for solving optimization problems. This is a key motivating driver for this work.

**Figure 9:**
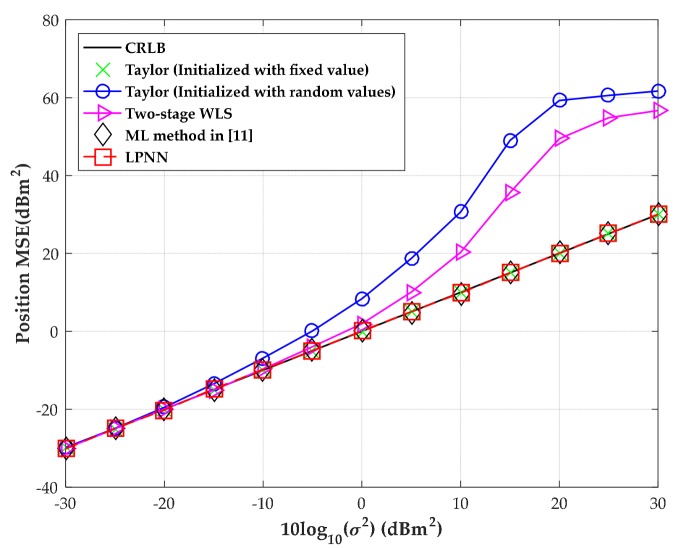
MSE versus level of measurement noise with randomly selected source positions.

The MSEs versus the level of measurement noise with moderate time deviation is shown in Figure 9; the source position is uniformly generated within a circle with radius 280 m centered on the origin in the 1000 independent runs. The range deviation variance is fixed at 400 m^2^. The outstanding performance of the LPNN method is verified in Figure 9.

**Figure 10:**
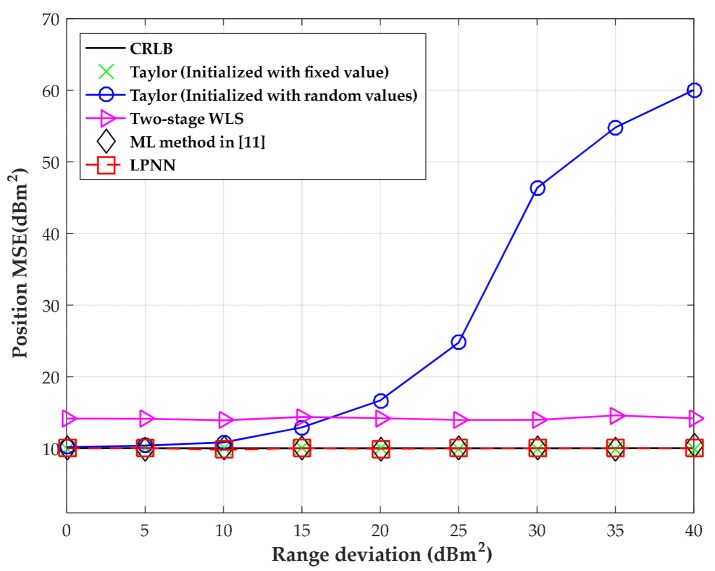
MSE versus level of time asynchronization with the source position located at (300, −280) m.

The MSEs versus the level of time asynchronization with moderate measurement noise $\sigma^{2}=10{\;m}^{2}$ is shown in Figure 10. We again see the optimality of the proposed LPNN method and its robustness in resisting the clock asynchronization even at a large range deviation, as the ML method in [11]. The other two numerical algorithms fail to yield an accurate estimate when the range deviation is large.

**Figure 11:**
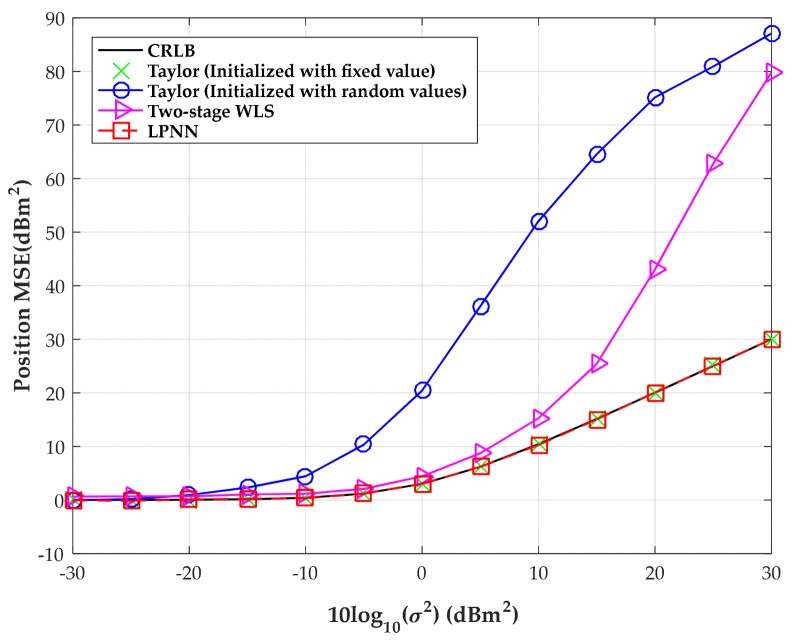
MSE versus level of measurement noise with the source position located at (300, −280) m.

In the second test, the effect of sensor location error is investigated and the range deviation variance is fixed at 400 m^2^ here. Four sensors are employed as in the first test. Figure 11 shows the MSEs versus the level of measurement noise under mild sensor location errors, namely $\sigma_{s}^{2}=1\;m^{2}$. It is observed from the figure that the proposed analog neural method outperforms the randomly initialized Taylor algorithm and two-stage WLS algorithm in a large measurement noise range.

**Figure 12:**
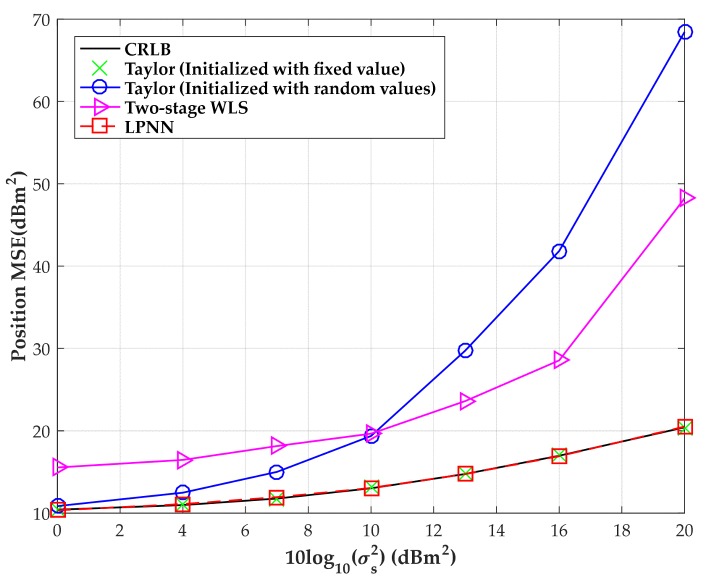
MSE versus level of sensor location error with the source position located at (300, −280) m.

The MSEs versus the sensor location error variance $\sigma_{s}^{2}\in [1,100]\;m^{2}$ with $\sigma^{2}=10\;m^{2}$ are exhibited in Figure 12. The figure shows that, under large measurement noise condition, the traditional Taylor algorithm and two-stage WLS algorithm fail to yield a reasonable estimation. It can also be noticed that the LPNN method is robust enough to achieve a better performance than other algorithms for a wide range of sensor position uncertainty levels.

In the preliminary formulation in Section 3 and Section 4, we assume that the measurement noise powers at different sensors are identical, and the sensor location errors at different sensors also have identical covariance. However, in practice, due to the different distances between the sensors and the source, the measurement noise covariance at different sensors will not be identical. Meanwhile, different sensors may have different location error covariance due to various influencing factors.

**Figure 13:**
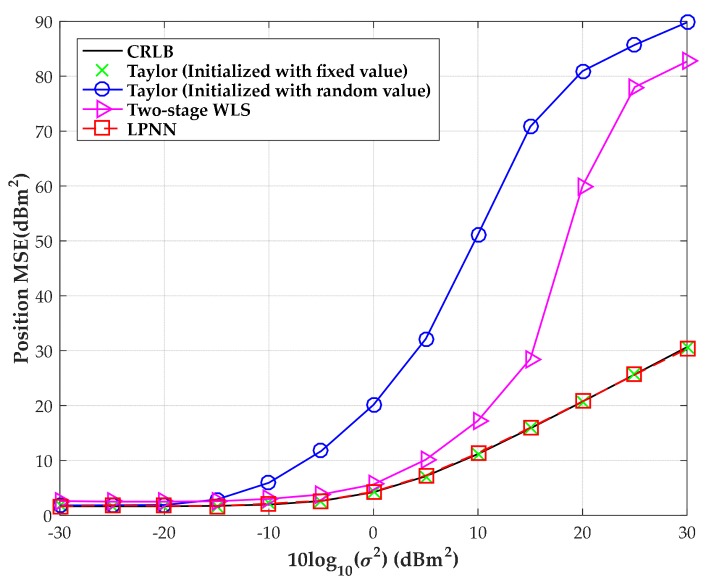
MSE versus level of measurement noise with the source position located at (300, −280) m.

Therefore, in the third test, four sensors are employed as in the second test and the measurement noise covariance at the four sensors are set as $\sigma_{1}^{2}\;=\;1.2\sigma^{2}$, $\sigma_{2}^{2}\;=\;\sigma^{2}$, $\sigma_{3}^{2}\;=\;1.3\sigma^{2}$, $\sigma_{4}^{2}=1.2\sigma^{2}$, respectively. The sensor location errors at the four sensors are set as $\sigma_{s,1}^{2}=\sigma_{s}^{2}$, $\sigma_{s,2}^{2}=2\sigma_{s}^{2}$, $\sigma_{s,3}^{2}=1.5\sigma_{s}^{2}$, $\sigma_{s,4}^{2}=1.3\sigma_{s}^{2}$, respectively. The range deviation variance is fixed at 400 m^2^ here. Figure 13 shows the MSEs versus the level of measurement noise under mild sensor location errors, namely $\sigma_{s}^{2}=1\;m^{2}$. It can be observed from Figure 13 that the proposed LPNN method can still yield better estimation results and attain CRLB even when the sensors have different measurement noise powers, as well as different location error covariance.

In the fourth test, we examine the sensitivity to the initial value of the proposed method and the iterative Taylor method and ML method in [11] through simulation. The positions of four sensors are the same in the first test and the true source position is located at (30, −20) m . Here, we assume that the sensor positions are exactly known, which is consistent with the model established in Section 3. Therefore, the unknown parameters to be estimated are source position u and range deviation $\delta_{o}$. Besides, the measurement noise covariance is fixed at 0 dBm^2^ and the range deviation variance is set as $100\;m^{2}$.

**Figure 14:**
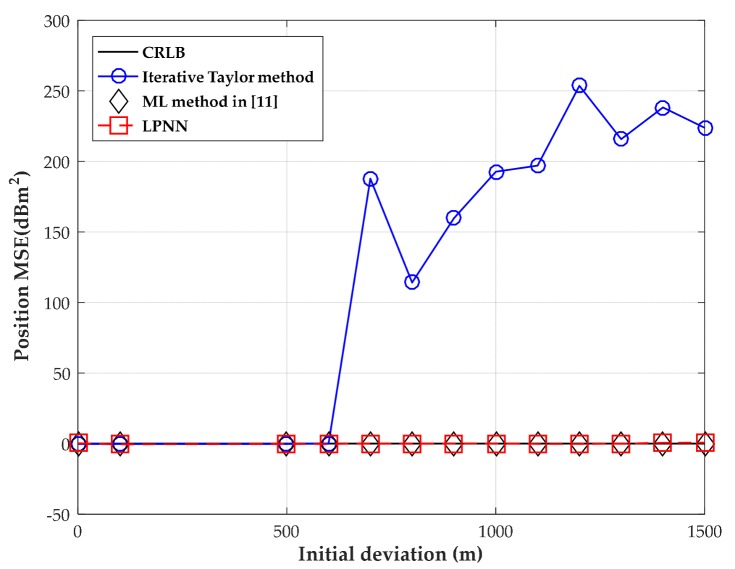
MSE versus level of initial deviation with the source position located at (30, −20) m.

In order to illustrate the influence of initial value selection on the estimation result, the initial value of u and $\delta_{o}$ is generated by:

(53) $$\left[\begin{array}{c} u_{\mathrm{ini}}\\ \delta_{o\mathrm{ini}} \end{array}\right]=\left[\begin{array}{c} u\\ \delta_{o} \end{array}\right]+\left[\begin{array}{c} \Delta u\\ \Delta \delta_{o} \end{array}\right]\cdot \Delta \;$$

where Δu and $\Delta \delta_{o}$ are randomly generated from (−1, 1). The initial deviation Δ is modified from 0 m to 1500 m to achieve different initial deviation. The corresponding MSEs versus the level of initial deviation Δ with moderate measurement noise and time offset are shown in Figure 14.

As shown in Figure 14, the iterative Taylor method fails when the initial value deviates from the true value, e.g., when Δ is over 600 m, because this method may converge to the local minima or even be unable to converge when it is not fine initialized. However, the proposed LPNN is robust enough to resist the impact of initial value as the ML method employed in [11] than the iterative Taylor method. We point out that the amount of deviation considered here is possible due to the fact that the source could lie at a random place and the range deviation $\delta_{o}$ is uncertain. The results also explain why the random-initialized Taylor method deviates the CRLB immediately in our paper.

Additionally, it is worth noting that the LPNN needs to be initialized because the initial state is necessary to be preset for solving differential equations. Since the LPNN network is a self-feedback system, it can eventually reach a steady state through feedback adjustment from an initial state within the attraction domain [16].

## 8. Conclusions

Based on the LPNN framework, we propose two analog neural network models for TOA-based source localization in the presence of clock asynchronization due to the non-cooperative source and sensor position uncertainties in this paper. Simulations demonstrate the superiority of the proposed method to the conventional numerical TOA-based positioning algorithms under different location geometries. Specifically, the proposed LPNN performs better even at large noise conditions and is robust enough to resist the impact of clock asynchronization and sensor position uncertainties compared with other numerical algorithms. Besides, simulation verifies that the proposed LPNN is insensitive to the initial value due to its self-feedback structure. What should also be mentioned is that the problems of clock offset and clock drift at the sensor, as well as the potential data association when there are multiple emitting sources, are not considered in this paper. These tasks are challenging but meaningful, and we will explore them in our future work.
